# Ageing genetic signature of hypersomatotropism

**DOI:** 10.1098/rsob.200265

**Published:** 2021-04-14

**Authors:** Abdalla Elbialy

**Affiliations:** Laboratory of Fish Diseases, Faculty of Veterinary Medicine, Damanhour University, Damanhour 22511, Egypt

**Keywords:** acromegaly, growth hormone, ageing signature, zebrafish

## Abstract

Acromegaly is a pathological condition that is caused by over-secretion of growth hormone (GH) and develops primarily from a pituitary adenoma. Excess GH exposure over a prolonged period of time leads to a wide range of systemic manifestations and comorbidities. Studying the effect of excess GH on the cellular level could help to understand the underlying causes of acromegaly health complications and comorbidities. In our previous publications, we have shown that excess GH reduces body side population (SP) stem cells and induces signs of premature ageing in an acromegaly zebrafish model. Here, we study acromegaly ageing in greater depth at the level of gene expression. We investigated whether acromegaly induces an ageing genetic signature in different organs. Using the GenAge database, our acromegaly model showed a significant enrichment of ageing genetic datasets in the muscle but not in other organs. Likewise, the hierarchical clustering of wild type (WT), acromegaly and aged RNA data from various organs revealed the similarity of gene expression profiles between the acromegaly and the aged muscles. We therefore identified overlapping differentially expressed genes (DEGs) in different organs between acromegaly and aged zebrafish. Importantly, about half of the muscle, liver and brain acromegaly DEGs overlapped with aged zebrafish DEGs. Interestingly, overlapping was observed in the same way; acromegaly-up DEGs overlapped with aged zebrafish up DEGs, not down DEGs, and *vice versa*. We then identified the biological functions of overlapping DEGs. Enrichment database analysis and gene ontology showed that most overlapping muscle genes were involved in ageing metabolism, while overlapping liver DEGs were involved in metabolic pathways, response to hypoxia and endoplasmic reticulum stress. Thus, this study provides a full ageing genetic signature of acromegaly at the gene expression level.

## Graphical abstract

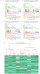

## 1. Introduction

Acromegaly is a progressive disease resulting from excess growth hormone (GH) levels and, subsequently, insulin-like growth factor 1 (IGF-1). Acromegaly is usually caused by a pituitary adenoma. In fewer cases, the disease may be caused by ectopic GH secretion or by pituitary hyperplasia [1]. Gigantism and acromegaly are both rare disorders caused by excess GH and IGF-1 secretions; however, gigantism occurs when GH excess triggers linear development prior to puberty, whereas acromegaly occurs after epiphyseal closure [2].

Excessive exposure to GH causes somatic disfigurement, including broad arms, legs, thickened soft tissues, enlarged face, nose and bulging of the forehead. Progressive acromegaly forms are characterized by skeletal defects leading to dorsal kyphosis and distortion of the rib cage [1,3].

In addition to abnormal soft-tissue growth and hypertrophy, patients with acromegaly develop a number of systemic manifestations and comorbidities, including gastrointestinal disease, reproductive disorders, arthritis, carpal tunnel syndrome, weakness, colon polyps, diabetes mellitus, kidney disorders, neuropathy and cardiovascular disease [4,5].

Over the past two decades, we have gained a better understanding of the genetic aetiology of gigantism and acromegaly. Several novel gene mutations that disrupt intracellular somatotrophic pathways to mediate GH hypersecretion have been identified, including aryl hydrocarbon receptor interacting protein (AIP) gene germline mutations [6] and X-linked acrogigantism syndrome (XLAG) trait [7].

Systemic manifestations of acromegaly are widespread throughout the body, suggesting that the pathological effect of excess GH acts on the cellular level. The discovery of the underlying genetic causes of acromegaly is crucial for the development of new diagnostic and therapeutic approaches. Less research, however, is focused on the pathological impact of excess GH and how GH works at the cellular level to cause acromegaly comorbidities.

In our previous publications, we developed a GH oversecreting zebrafish model of acromegaly to investigate the pathological effects of excess GH on cell biology. Many disrupted parameters have been identified in acromegaly somatic and stem cells, such as increased DNA damage and impaired DNA repair pathways [3,8]. Among the major disorders in our acromegaly model owing to excess GH include a progressive decrease in the number of side population (SP) stem cells in the body and an increase in oxidative stress in stem cells. In addition, we have observed signs of premature ageing [8]. Here, therefore, we study acromegaly ageing more deeply at the level of gene expression by examining whether acromegaly exhibits enrichment of ageing genetic signatures in various organs. In addition, we investigate whether there is an overlap between acromegaly and ageing differentially expressed genes (DEGs) and identify shared biological themes between acromegaly and ageing.

## 2. Results

### 2.1. Acromegaly muscle ageing at gene expression level

In our previous publications, a tilapia-GH overexpressing zebrafish acromegaly model [3] was produced to study the pathological effects of excess GH on cell biology. We have identified many disrupted signalling pathways and biological processes at the cellular level. Among the main findings was a decline in the number of stem cells and premature signs of ageing in our model [3,8]. In order to investigate ageing in acromegaly at the level of gene expression, we used the GenAge database (https://genomics.senescence.info/genes/index.html), which was developed from meta-analysis studies to identify the common genetic signature of ageing in different organs. We used our previously published RNA seq data of mRNA extracted from the liver, muscle, kidney and brain of wild type (WT) (1-year-old), acromegaly (1-year-old) and aged zebrafish (3-year-old) (RNA seq data available at Gene expression omnibus (GEO) accession no. GSE113169 [9]). Gene set enrichment analysis (GSEA) was then performed using acromegaly RNA seq data from the liver, muscle, kidney and brain to test for enrichment of GenAge genetic signature genes in corresponding organs.

**Figure 1.**
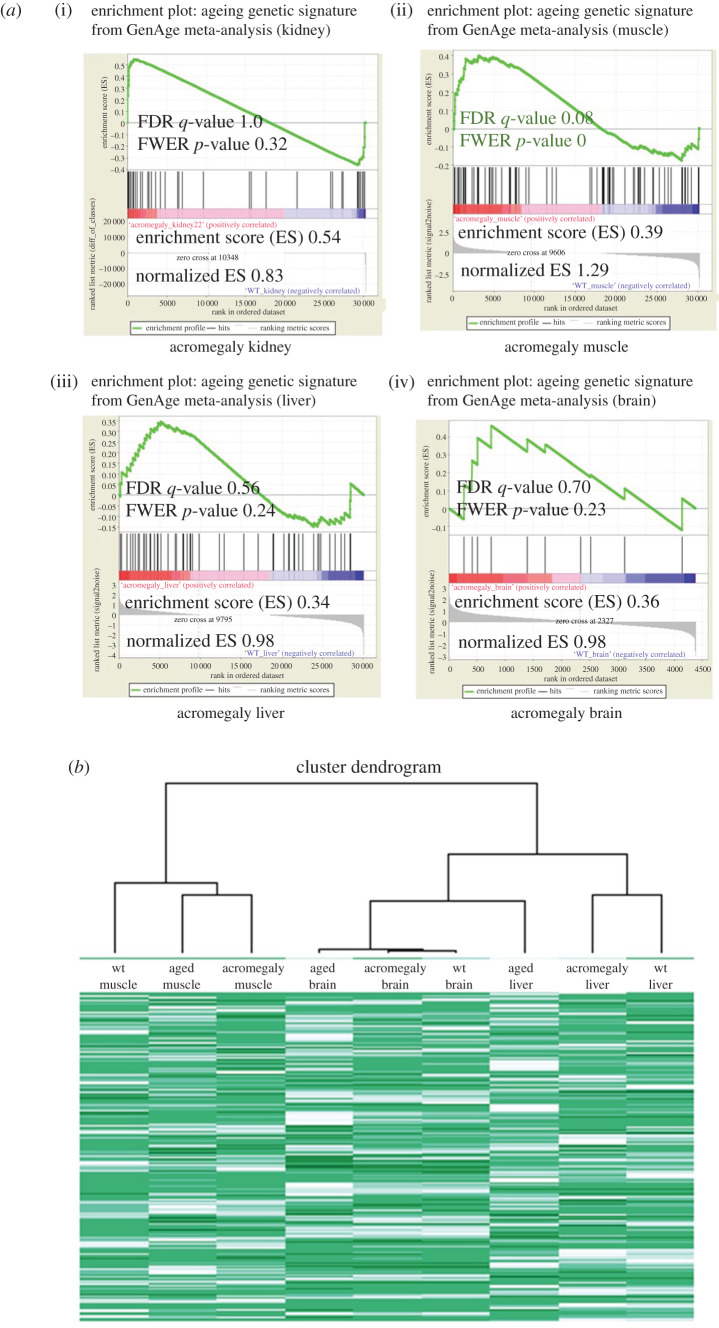
Acromegaly muscle ageing at gene expression level. (*a*) GSEA results of ageing signature genes from the GenAge database in the acromegaly kidney, muscle, liver and brain. Significant *p*-values less than 0.05 and FDR *q*-values less than 0.25 are shown in red. (*b*) Hierarchical cluster analysis dendrogram and heatmap of RNA seq data from the muscle, liver and brains of WT, acromegaly model (1-year-old) and aged zebrafish (3-year-old).

As we can see in figure 1*a*, the enrichment scores of ageing genetic datasets were positive in all the acromegaly organs, indicating upregulation of the GenAge gene sets in the respective organs; however, the enrichment was significant only in the acromegaly muscles (*p*-value < 0.01 and false discovery rate (FDR) *q*-value = 0.08).

In order to confirm these results, we investigated acromegaly ageing at the level of gene expression using a different approach. We generated a hierarchical cluster analysis dendrogram of RNA seq data from the muscle, liver and brains of WT, acromegaly model (1-year-old) and aged zebrafish (3-year-old) (figure 1*b*). Importantly, the hierarchical clustering yielded the same results and showed similarities in patterns of gene expression between acromegaly and aged zebrafish only in the muscle, but not in the liver or brain (figure 1*b*) [8].

### 2.2. Overlaping differentially expressed genes between acromegaly and aged zebrafish

Adj *p*-value was used to identify the DEGs of acromegaly (1-year-old) and aged zebrafish (3-year-old) in different organs relative to WT samples (alpha = adj *p*-value < 0.05).

**Figure 2.**
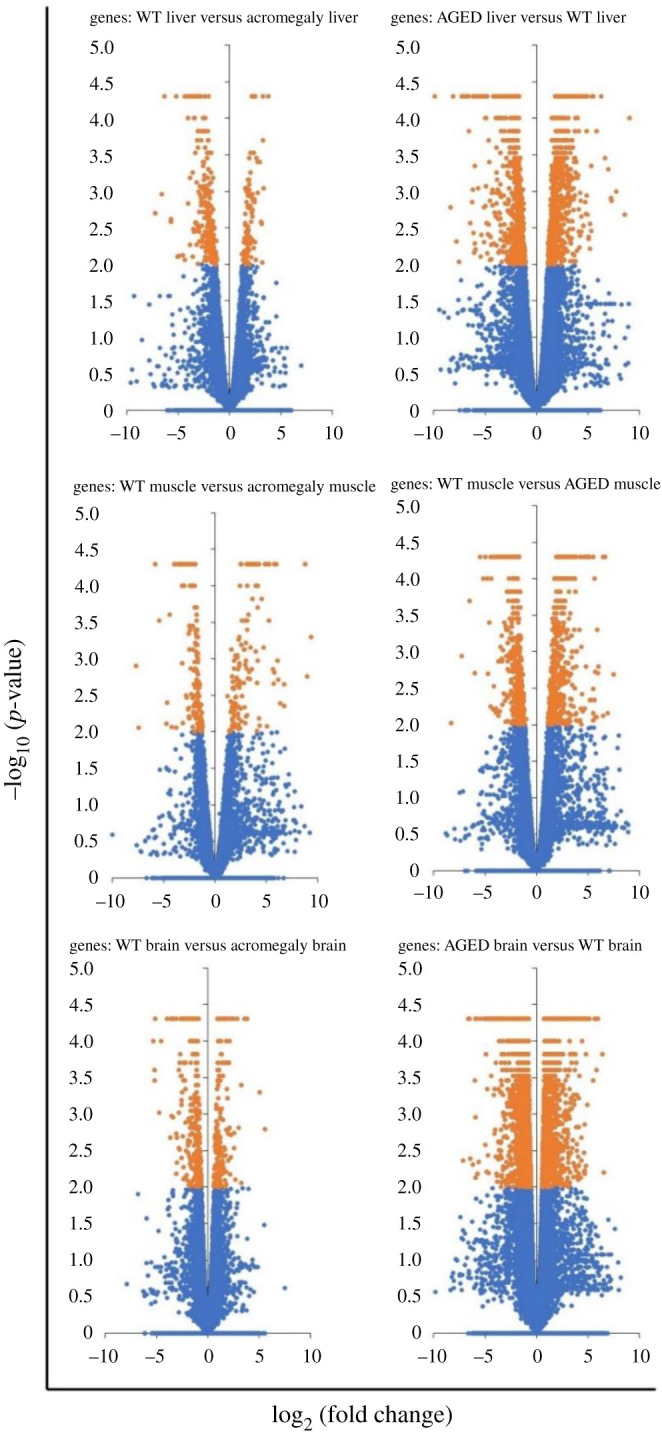
Volcano plots of acromegaly and aged muscle genes versus WT muscle. *Y*-axis, −log10 (*p*-value); *X*-axis, log_2_ (fold change). Orange coloured genes are considered significant (*n* = 3).

Figure 2 displays the volcano plots of the RNA seq genes of the acromegaly and the aged muscle, liver and brain.

Since our acromegaly model showed signs of premature ageing and similar patterns of gene expression to the aged muscle [3], we investigated whether acromegaly DEGs overlap with DEGs of aged zebrafish. We established Venn diagrams of up and down DEGs from acromegaly and aged muscle, liver and brain.

**Figure 3.**
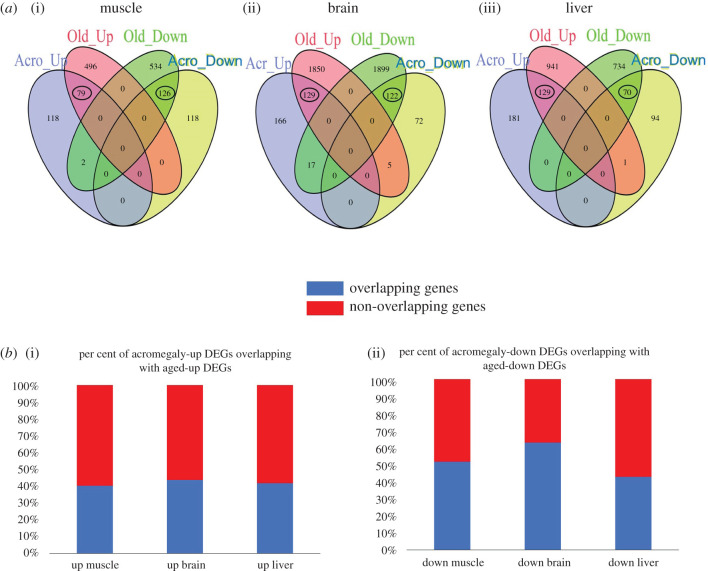
Acromegaly overlap DEGs. (*a*) Venn diagrams of up-and-down DEGs of acromegaly and aged zebrafish muscle, brain and liver. (*b*) Bar chart showing the percentage of up-and-down acromegaly DEGs overlapping with aged zebrafish.

Although the number of DEGs in aged zebrafish was higher, most of the acromegaly DEGs overlapped with aged DEGs (figures 2 and 3*a*). It is important to note that overlapping has been observed in the same way; acromegaly-up DEGs overlapped with aged-up DEGs, not down DEGs, and *vice versa* (figure 3*a*).

More than 50 per cent of acromegaly-down DEGs overlapped with aged-down DEGs in the muscle, while about 40 per cent of up DEGs overlapped with aged-up DEGs (figure 3*b*). Importantly, there was almost no overlap between acromegaly-down DEGs and aged-up DEGs or between acromegaly-up DEGs and aged-down DEGs in the muscle (figure 3*a*). These results support our previous observation that the acromegaly muscles show similarity in the patterns of gene expression to aged zebrafish.

Although hierarchical clustering and GSEA results (figure 1) did not show ageing in the acromegaly brain, the Venn diagram revealed that 65% of acromegaly-down DEGs overlapped with aged-down DEGs in the brain. And about 40% of up DEGs overlapped in aged DEGs (figure 3). Interestingly, the same results were observed in the liver (figure 3).

### 2.3. Enriched biological themes and signalling pathways of overlapping DEGs in muscle.

**Figure 4.**
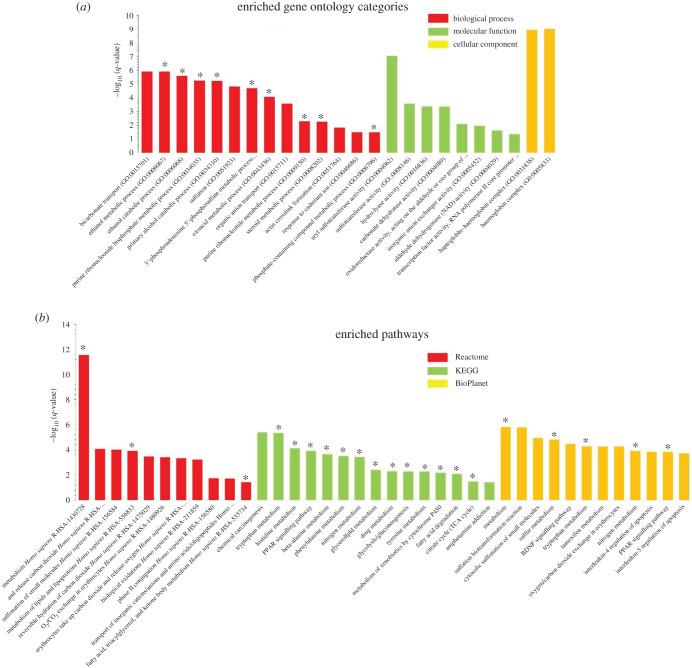
Enriched GO and pathway analysis of muscle overlapping DEGs. (*a*) GO analysis showing enriched biological processes, molecular functions and cellular components of muscle overlapping DEGs. (*b*) Histogram for enriched signalling pathways of muscle overlapping DEGs from Reactome, KEGG and BioPlanet databases. *Y*-axis, the statistical significance of the enrichment; *X*-axis, pathway categories. Metabolism-related pathways and GO categories are denoted by asterisks. BDNF, brain-derived neurotrophic factor; PPAR, peroxisome proliferator-activated receptor.

We therefore performed enrichment analysis of overlapping DEGs to identify the common signalling pathways and biological processes between acromegaly and ageing. Gene ontology (GO) analysis of overlapping DEGs between acromegaly and aged muscle revealed enrichment of metabolic pathways such as ethanol, oxoacid and steroid metabolism in biological processes (figure 4*a*), and enrichment of enzymatic activities for molecular function (figure 4*a*). We used the KEGG database (https://www.genome.jp/kegg/) to classify the roles of enriched enzymatic activities. The analysis showed that most of the enriched enzymatic activities are related to metabolic processes (table 1), while cellular component analysis revealed enrichment of the haptoglobin– haemoglobin complex (figure 4*a*).

**Table 1.** KEGG pathways of enriched enzymatic activities in figure 4*a*.

enriched metabolic function	related KEGG pathway
hydro-lyase activity	methane metabolism
	metabolic pathways
	microbial metabolism in diverse environments
carbonate dehydratase activity	nitrogen metabolism
	metabolic pathways
oxidoreductase activity	glycolysis/gluconeogenesis
	citrate cycle (TCA cycle)
	pyruvate metabolism
	nitrotoluene degradation
	propanoate metabolism
	butanoate metabolism
	methane metabolism
	carbon fixation pathways in prokaryotes
	metabolic pathways
	biosynthesis of secondary metabolites
	microbial metabolism in diverse environments
aldehyde dehydrogenase (NAD) activity	glycolysis/gluconeogenesis
	ascorbate and aldarate metabolism
	fatty acid degradation
	valine, leucine and isoleucine degradation, lysine degradation, arginine and proline metabolism
	histidine metabolism
	tryptophan metabolism
	beta-alanine metabolism
	glycerolipid metabolism
	pyruvate metabolism
	chloroalkane and chloroalkene degradation
	limonene and pinene degradation
	insect hormone biosynthesis
	metabolic pathways
	biosynthesis of secondary metabolites
	microbial metabolism in diverse environments

Interestingly, pathway analysis has shown that most of the enriched signalling pathways are also related to metabolism (figure 4*b*). Reactome database analysis (https://reactome.org/) revealed enrichment of metabolic pathways of lipids, phospholipids, fatty acid and triglycerides, while KEGG and BioPlanet database analysis (https://tripod.nih.gov/bioplanet/) showed enrichment of fatty acids, glycolysis, nitrogen and various amino acid metabolic pathways.

**Figure 5.**
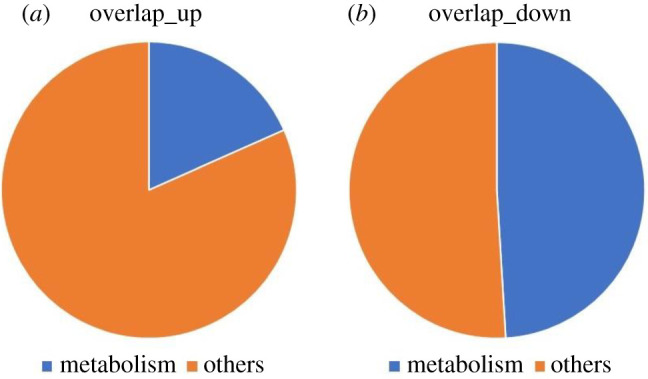
Pie chart showing the percentage of up- and down-regulated overlapping DEGs that are related to metabolic processes in muscle.

In order to investigate the percentage of overlapping DEGs involved in ageing muscle metabolism, we quantified the metabolic-related genes as a total number of overlapping genes. We have found that most of the metabolic-related overlapping DEGs are downregulated. Approximately 50% of the down overlapping DEGs and 20% of the up-overlapping DEGs are related to metabolism (figure 5).

### 2.4. Enriched biological themes and signalling pathways of overlapping DEGs in liver

The Venn diagram showed that about 40% of acromegaly liver DEGs overlapped with aged DEGs (figure 3). We therefore investigated the biological functions of these overlapping genes.

**Figure 6.**
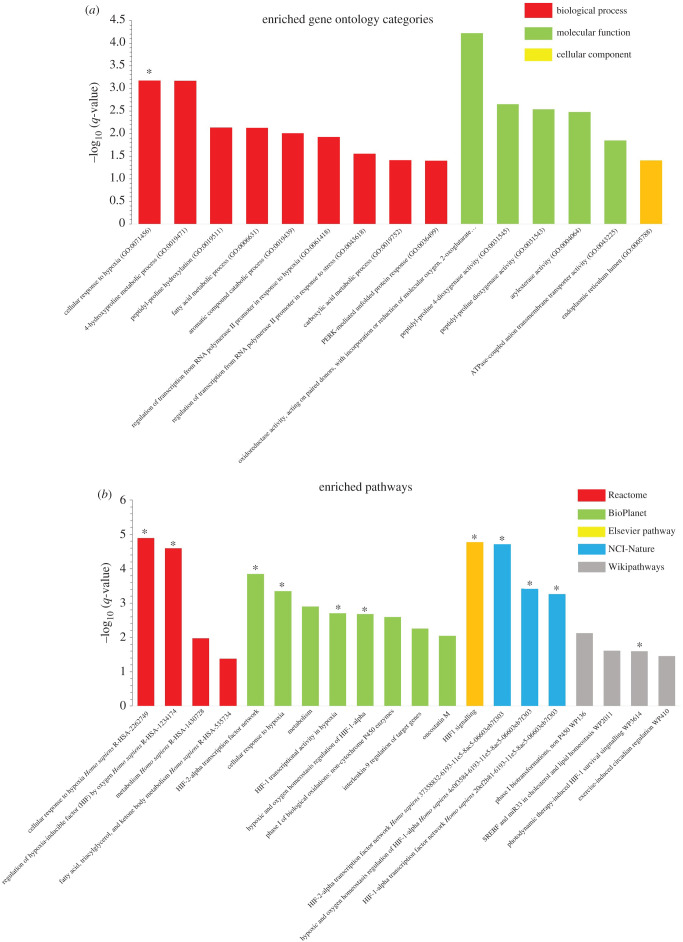
Enriched GO and pathway analysis of liver overlapping DEGs. (*a*) GO analysis showing enriched biological processes, molecular functions and cellular components of liver overlapping DEGs. (*b*) Histogram for enriched signalling pathways of liver overlapping DEGs from Reactome, Elsevier pathway, Wikipathways, NCI-Nature and BioPlanet databases. *Y*-axis, the statistical significance of the enrichment; *X*-axis, pathway categories. Hypoxia-related pathways and GO categories are denoted by asterisks. HIF-1, hypoxia-inducible factor; miR33, micro RNA identifier; SREBF, sterol regulatory element–binding protein gene.

GO analysis of overlapping DEGs in the liver revealed enrichment of three main categories: metabolic pathways, response to hypoxia and ER stress. Response to hypoxia, fatty acid metabolic process, regulation of transcription, protein kinase R (PKR)-like endoplasmic reticulum kinase (PERK)-mediated unfolded protein response and ER were among the enriched biological themes (figure 6*a*). Consistently, we have previously shown that ER stress induction is one of the main features of acromegaly (under publication).

**Figure 7.**
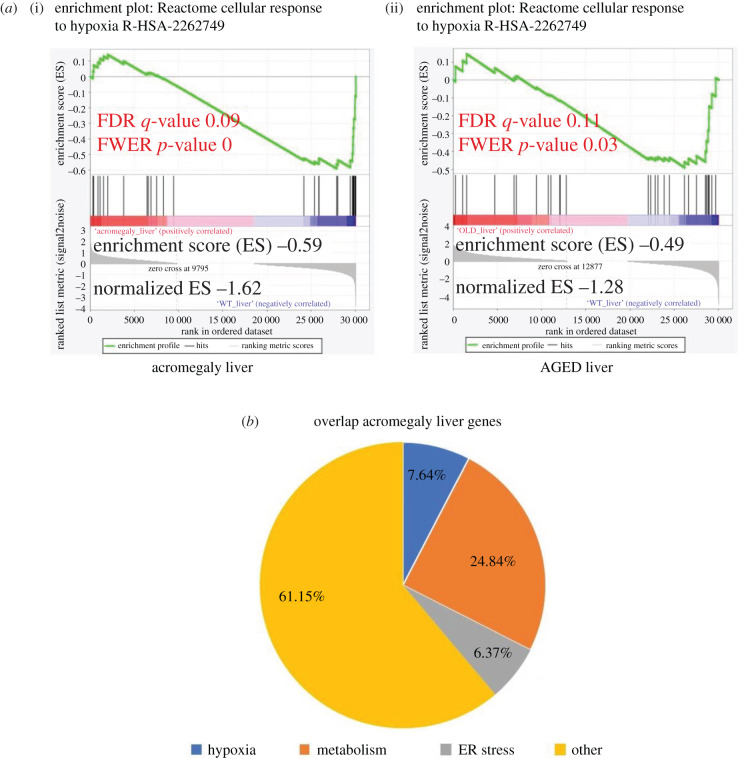
Hypoxia, metabolism and stress-related genes in overlapping liver DEGs. (*a*) Illustration of statistically significant GSEA results of cellular response to hypoxia. Significant *p*-values <0.05 and FDR *q*-values <0.25 are written in red. The reported *p*-value of 0.0 indicates an actual *p*-value of less than 0.01 (*n* = 3). (*b*) Pie chart showing the percentage of overlapping DEGs that are related to metabolism, hypoxia and ER stress in liver.

Similarly, pathway analysis from Reactome, Elsevier pathway (http://www.transgene.ru/disease-pathways/), Wikipathways (https://www.wikipathways.org/index.php/WikiPathways), NCI-Nature (http://pid.nci.nih.gov/) and BioPlanet databases showed enrichment of pathways related to hypoxia and metabolism (figure 6*b*). Importantly GSEA showed significant downregulation of response to hypoxia in the liver of acromegaly (1-year-old) and aged zebrafish (3-year-old) (figure 7*a*).

We therefore quantified overlapping genes related to metabolism, hypoxia response and ER stress as a total number of overlapping genes in the liver. We found that most overlapping genes in the liver are related to these three categories (figure 7*b*).

### 2.5. Enriched biological themes and signalling pathways of overlapping DEGs in brain

Similar to the muscle and liver, the Venn diagram showed that most acromegaly brain DEGs overlapped with the aged brain. As we can see in figure 3, approximately 65% of acromegaly-down DEGs overlapped with aged-down DEGs in the brain.

**Figure 8.**
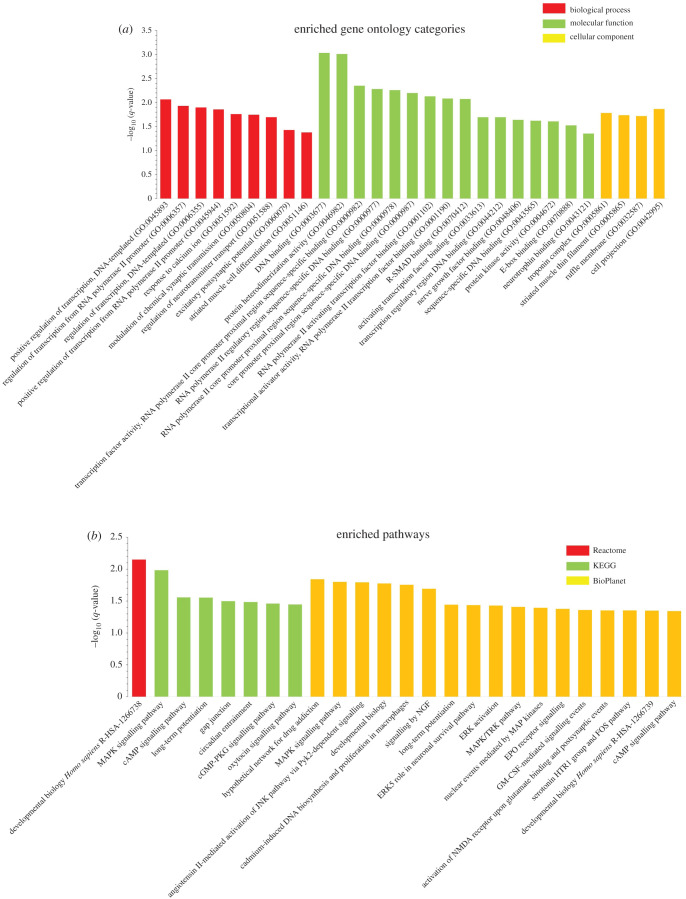
Enriched GO and pathway analysis of brain overlapping DEGs. (*a*) GO analysis showing enriched biological processes, molecular functions and cellular components of brain overlapping DEGs. (*b*) Histogram for enriched signalling pathways of brain overlapping DEGs from Reactome, KEGG and BioPlanet databases. *Y*-axis, the statistical significance of the enrichment; *X*-axis, pathway categories. cGMP-PKG, cyclic guanosine monophosphate-dependent protein kinase or protein kinase G; epo, erythropoietin; ERKs, extracellular signal-regulated kinases; FOS, Fos proto-oncogene; GM-CSF, granulocytemacrophage colony-stimulating factor; HTR1, 5-hydroxytryptamine receptor 1; JNK, c-Jun N-terminal kinase; MAPK, mitogen-activated protein kinase; NGF, nerve growth factor; NMDA, n-methyl-D-aspartate; R-SMADs, receptor-regulated SMADs; TRK, tropomyosin receptor kinase.

GO analysis of overlapping DEGs in the brain has shown that positive regulation of transcription, DNA binding, neurotrophins binding and regulation of neurotransmitter transport were enriched for biological process and molecular function. Troponin, ruffle membrane and cell projection were enriched for cellular components (figure 8*a*).

Pathway analysis from Reactome, KEGG and BioPlanet databases showed the enrichment of developmental biology, gap junction, circadian entrainment, long-term potentiation and MAPK signalling pathway (figure 8*b*).

### 2.6. Growth hormone-cultured human oocytes showing ageing at gene expression level

In this study and our previous publications, we have shown that excess GH influenced ageing and stem cell number and integrity in our acromegaly zebrafish model. It is therefore important to investigate whether excess GH could have the same effect on human cells. Here, we have tried to study the ageing signature of GH-treated human cells available at GEO (gene expression omnibus). We retrieved single-cell RNA seq data of human oocyte cultured by GH (GEO accession no. GSE133161).

**Figure 9.**
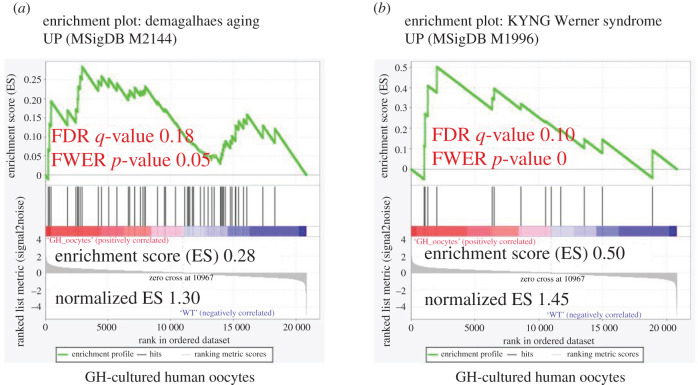
GH-cultured human oocytes showing ageing at gene expression level. (*a*) Illustration of statistically significant GSEA results against a geneset that consistently overexpressed with age, based on meta-analysis of microarray data (MSigDB no. M2144) and (*b*) premature ageing disorder dataset (Werner syndrome, MSigDB no. M1996). Significant *p*-values < 0.05 and FDR *q*-values < 0.25 are written in red. The reported *p*-value of 0.0 indicates an actual *p*-value of less than 0.01.

We used GSEA analysis to investigate whether GH oocytes enrich ageing signatures. Interestingly, this gene set and a dataset for the premature ageing disorder (Werner Syndrome, MSigDB no. M1996) were significantly upregulated in GH-cultured human oocytes (figure 9).

## 3. Discussion

Zebrafish are a promising model for the study of human genetic diseases owing to the availability of a fully sequenced genome, ease of genetic manipulation, transparency of larvae, short life cycle and low housing costs. In addition, zebrafish are physiologically similar to mammals [10].

In an attempt to identify the molecular mechanisms underlying acromegaly comorbidities, a tilapia-GH overexpressing zebrafish was developed as an acromegaly model. We have shown that excess GH influences cellular integrity throughout the body [3,8]. Furthermore, we identified many disrupted biological parameters in acromegaly somatic and stem cells. Interestingly, our zebrafish model showed signs of premature ageing and reduced SP stem cells in the kidneys, muscles and brains [3,8]. That encouraged us to study acromegaly ageing more deeply at the level of gene expression.

GenAge is a human and model animal database for ageing and longevity. GenAge datasets used in this research were derived from meta-analysis studies to detect the ageing genetic signature of every organ [11]. Importantly, both the GSEA analysis against GenAge sets and the hierarchical clustering of RNA seq genes showed enrichment of ageing in acromegaly muscles.

It is important to mention that the aged zebrafish used in this study are very aged zebrafish (3 years old).

Although the enrichment of the GSEA analysis in other organs was not significant (by *p*-value), the enrichment scores were positive for all the acromegaly organs tested, indicating the upregulation of the ageing gene sets in the respective organs.

The association of acromegaly gene expression pattern towards ageing was more prevalent in the results of the Venn diagram between aged and acromegaly DEGs. Approximately half of the acromegaly DEGs have been enriched in aged zebrafish. Interestingly, DEGs overlapping has been observed in the same way in all organs tested; acromegaly-up DEGs overlapped with aged-up DEGs, not aged-down DEGs, and *vice versa*. It is important to note that most overlapping DEGs were downregulated in the muscle and upregulated in the liver.

Using overlapping DEGs, we identified the Shared biological themes between acromegaly and ageing in each organ. In the liver, acromegaly shared three main categories: response to hypoxia, ER stress and some metabolic pathways, while in the muscle, most overlapping DEGs were involved in ageing metabolism. Consistently, we have previously shown that both acromegaly and ageing trigger ER stress (under publication).

In this study and our previous publications, we have shown that excess GH influenced both ageing and stem cell number and integrity in a zebrafish acromegaly model. However, these are preclinical studies and therefore it is important to study the ageing and integrity of the stem cells in the different tissues of acromegaly patients. Although we have shown that GH-cultured human oocytes enrich ageing genetic signatures, clinical studies in acromegaly patients are required to investigate the effects of excess GH on ageing and stem cells in all organs.

GH effects are mainly mediated by the GH/IGF-1 axis, which is the main controller of somatic growth. IGF-1 level is used more often for routine diagnosis of acromegaly as it is stable during the day [5]. In our previous publication, it has been shown that the IGF-1 acromegaly model is comparable to the IGF-1 acromegaly level [3]. Additionally, our acromegaly model and patients with acromegaly exhibited DNA damage induction. Previous studies have reported the induction of DNA damage to peripheral lymphocytes in acromegaly [12,13], while our model has induced DNA damage in various organs [3]. Thus, our acromegaly zebrafish model showed similarities in many biological parameters with acromegaly patients.

Moreover, similar to acromegaly patients, GH transgenic rats and GH transgenic salmon, our model showed substantial induction of oxidative stress [3,8,12,14,15].

Oxidative stress and DNA damage are major players in ageing and age-related diseases [16].

Acromegaly patients develop some age-related health complications such as muscle weakness, diabetes mellitus, neuropathy, reproductive disorders, arthritis, kidney disorders, neuropathy and cardiovascular disease. According to this study and our previous studies, these health complications could be attributed, at least in part, to the contribution of ageing in acromegaly.

The contribution of ageing to acromegaly health complications can provide a deeper understanding of acromegaly systemic manifestations, the pathological effects of excess GH could help identify potential targets for therapeutic interventions.

According to our analysis, the only difference identified between our model and acromegaly patients is the ubiquitous expression of GH in the zebrafish acromegaly model, whereas, in acromegaly patients, GH is primarily secreted from a pituitary adenoma [3].

## 4. Methods

### 4.1. Data

We used our RNA seq data available at Gene expression omnibus (GEO) accession nos. GSE113169, GSE153755. We used RNA seq data of acromegaly, WT (1-year-old) and aged zebrafish (3-year-old) from muscle, brain, liver and kidney. DEGs were detected previously [3,8] using cummerbund r package (https://www.bioconductor.org/packages/release/bioc/html/cummeRbund.html) (*α* = adj, *p*-value < 0.05).

### 4.2. Data analysis

#### 4.2.1. Hierarchical clustering and heatmap

Hierarchical clustering of RNA seq data from muscle, liver and kidney of acromegaly, WT and aged zebrafish was conducted using the heatmap.2 function of gplots r package. (https://rdrr.io/cran/gplots/) [9]

#### 4.2.2. Gene set enrichment analysis

First, we used Biomart (http://www.ensembl.org/biomart/martview) to convert the differentially expressed genes to their human orthologues, then we tested GSEA [17] for enrichment against the ageing genetic signature of the GenAge gene sets (https://genomics.senescence.info/genes/), Reactome cellular response to hypoxia (R-HAS-2262749), demagalhaes ageing UP (MSigDB no. M2144) and KYNG Werner syndrome (MSigDB no. M1996).

Gene sets were considered significant according to *p*-value < 0.05.

#### 4.2.3. Volcano plot

Volcano plots of acromegaly and aged muscle genes versus WT muscle were conducted using Microsoft Excel software.

*Y*-axis, −log_10_ (*p*-value); *X*-axis, log_2_ (fold change).

#### 4.2.4. Venn diagram

We used the VennDiagram R software package to construct a Venn diagram to identify overlapping genes between acromegaly muscle DEGs and aged muscle DEGs.

#### 4.2.5. Enriched pathway analysis

After converting the overlapping genes between acromegaly and aged zebrafish muscle to their human orthologues using the BioMart platform [18], we used the ENRICHR database to identify enriched signalling pathways.

ENRICHR pathways were considered significant according to *p*-value < 0.05

#### 4.2.6. Gene ontology

We used GO platform (http://geneontology.org/) to detect enriched biological themes of overlapping genes.

GO biological themes were considered significant according to *p*-value < 0.05

### 4.3. Statistical analysis

All experiments were performed on biological replicates. The size of the sample is reported in the relevant figure legends. Data were considered statistically significant if *p*-value < 0.05.
